# Fabrication and Characterization of Human Serum Albumin Particles Loaded with Non-Sericin Extract Obtained from Silk Cocoon as a Carrier System for Hydrophobic Substances

**DOI:** 10.3390/polym13030334

**Published:** 2021-01-21

**Authors:** Kanyaluck Jantakee, Ausanai Prapan, Saranya Chaiwaree, Nittiya Suwannasom, Waraporn Kaewprayoon, Radostina Georgieva, Yingmanee Tragoolpua, Hans Bäumler

**Affiliations:** 1 Department of Biology, Faculty of Science, Chiang Mai University, Chiang Mai 50200, Thailand; kanyaluckjan@gmail.com; 2 The Graduate School, Chiang Mai University, Chiang Mai 50200, Thailand; 3 Department of Radiological Technology, Faculty of Allied Health Sciences, Naresuan University, Phitsanulok 65000, Thailand; ausanaip@nu.ac.th; 4 Faculty of Pharmacy, Payap University, Chiang Mai 50000, Thailand; mam.chaiwaree@gmail.com (S.C.); waraporn.kpy@gmail.com (W.K.); 5 School of Medical Sciences, University of Phayao, Phayao 56000, Thailand; nittiya.su@up.ac.th; 6 Charité, Universitätsmedizin Berlin, Institute of Transfusion Medicine, 10117 Berlin, Germany; radostina.georgieva@charite.de; 7 Department of Medical Physics, Biophysics and Radiology, Medical Faculty, Trakia University, 6000 Stara Zagora, Bulgaria; 8 Research Center in Bioresources for Agriculture, Industry, and Medicine, Faculty of Science, Chiang Mai University, Chiang Mai 50200, Thailand

**Keywords:** non-sericin, human serum albumin (HSA), CCD-technique, A549 cell line

## Abstract

Non-sericin (NS) extract was produced from the ethanolic extract of *Bombyx mori* silk cocoons. This extract is composed of both carotenoids and flavonoids. Many of these compounds are composed of substances of poor aqueous solubility. Thus, this study focused on the development of a carrier system created from biocompatible and biodegradable materials to improve the biological activity of NS extracts. Accordingly, NS was incorporated into human serum albumin template particles with MnCO_3_ (NS-HSA MPs) by loading NS into the preformed HAS-MnCO_3_ microparticles using the coprecipitation crosslinking dissolution technique (CCD-technique). After crosslinking and template dissolution steps, the NS loaded HSA particles are negatively charged, have a size ranging from 0.8 to 0.9 µm, and are peanut shaped. The degree of encapsulation efficiency ranged from 7% to 57% depending on the initial NS concentration and the steps of adsorption. In addition, NS-HSA MPs were taken up by human lung adenocarcinoma (A549 cell) for 24 h. The promotion of cellular uptake was evaluated by flow cytometry and the results produced 99% fluorescent stained cells. Moreover, the results from CLSM and 3D fluorescence imaging confirmed particle localization in the cells. Interestingly, NS-HSA MPs could not induce inflammation through nitric oxide production from macrophage RAW264.7 cells. This is the first study involving the loading of non-sericin extracts into HSA MPs by CCD technique to enhance the bioavailability and biological effects of NS. Therefore, HSA MPs could be utilized as a carrier system for hydrophobic substances targeting cells with albumin receptors.

## 1. Introduction

Silkworm (*Bombyx mori*) is an important insect of great economic significance in the textile industry. Additionally, its pupa has been consumed for generations as a traditional food. Silk proteins, sericin and fibroin are natural macromolecule proteins derived from the outer shell of the cocoon. Silk is composed of 70% fibroin, 25% sericin, and 5% non-sericin in terms of the total cocoon shell weight. Carbohydrates, wax, flavonoids, and pigments are the key components of non-sericin in silk cocoons, and they act as a waterproof protective layer of the cocoon. Additionally, it was described that sericin proteins show antibacterial, antioxidative, and anti-inflammatory effects and can inhibit tumor growth and tyrosinase enzyme [1,2]. The previous study investigated the sericin mixture that was prepared using the hydro-lysate method by degumming the silk cocoon shell to identify any proteins that may possess a wide range of molecular mass. The biological activities of the sericin mixture are a result of the presence of sericin proteins or a combined effect of the non-sericin components. The natural coloring of a silk cocoon occurs on the surface of the sericin layer, which is dependent upon the strain of the *B. mori* silkworm [3]. The cocoons can appear in a range of colors including yellow, pink, golden-yellow, flesh, sasa (yellowish-green), and green. The yellow, pink, golden-yellow, and flesh pigments of the cocoons are derived from carotenoids that are known to be ether soluble components. Furthermore, the sasa and green pigments occur as a result of flavonoids that are known to be either aqueous soluble substances or hydrophilic substances [3,4,5]. In addition, a variety of cocoon shell colors may appear as a result of certain substances found in mulberry tree strains that occur during the feeding of *B. mori* larvae [6]. Biological functions, such as antioxidant activity and antityrosinase activity, have been studied in terms of their non-sericin components [7]. However, there are some limitations of these non-sericin components as they may contain components of poor aqueous solubility that can hinder their effects and bioavailability. Therefore, it is necessary to develop a carrier system to challenge their insolubility and to improve the biological effects of these non-sericin components for broader applications and usage.

Carrier systems are recognized as an effective approach for the delivery of bioactive compounds or drugs to targeted sites. At present, drug carrier systems have been used worldwide for the treatment of cancer, diabetes, allergies, infections, and inflammation, and they are now being adapted for other treatments [8]. Carrier particles can be classified by size and morphology or whether they are inorganic, polymeric, solid lipid, liposomes, nanocrystals, nanotubes, or dendrimers [9,10]. Nano and microstructure particles that have been established in the drug delivery system are achieved through polymers, such as polyhydroxyalkanoates (PHAs), poly-(lactic-co-glycolic acid (PLGA), cyclodextrins (CDs)) [11], and biopolymer based particles including protein/polypeptide, silk fiber, collagen and gelatin, β-casein, Zein, polysaccharides, albumin, and others [8,12].

Human serum albumin (HSA) is a natural biopolymer that has gained attention for its role in alternative drug delivery systems in biomedical field. This is because albumin is the most abundant form of plasma protein found in human blood, and it serves as a biocompatible and biodegradable carrier. Moreover, albumin is highly water-soluble, has a long half-life and contains several hydrophobic binding pockets that provide a good range of protein particles. These HSA particles also display nontoxic and nonimmunogenic properties [13,14]. Additionally, albumin-based micelle nanoparticles have been reported to serve as effective carriers for hydrophobic drugs such as doxorubicin [15,16]. Further, HSA-based nanoparticles have been exploited for their potential as beneficial products in various diagnoses and prescribed therapies, such as Levemir^®^ and Victoza^®^ in the treatment of diabetes, Abraxane^®^ for treating solid tumors, and ^99m^Tc-aggregated albumin for diagnostic use in nuclear medicine [13].

The incorporation of hydrophobic compounds into HSA particles can increase their degree of stability, solubility, and biological activity. HSA MPs can be fabricated by the coprecipitation crosslinking dissolution technique (CCD-technique) [17,18,19]. Firstly, coprecipitation is performed by mixing HSA-containing MnCl_2_ and Na_2_CO_3_ solutions. Subsequently, HSA was cross-linked within the template by means of glutaraldehyde. After dissolution of the template with ethylene diamine tetra-acetic acid solution (EDTA), pure HSA-MPs were obtained. The uniform size of the peanut-shaped particles was achieved in a submicron size with high protein entrapment efficiency [19]. Thus, these particles were found to be suitable for the loading of bioactive compounds [17], while the CCD technique demonstrated reasonable high drug-loading capacity.

Consequently, HSA MPs were designed in this study for the delivery of hydropho-bic substances and non-sericin extracts (NS) using the CCD-technique. The cellular uptake by the A549 cell line of these particles and the effect of NS-HSA MPs on macrophage cells were investigated.

## 2. Materials and Methods 

Glutaraldehyde (GA), fluorescein isothiocyanate (FITC), manganese chloride tetra-hydrate (MnCl_2_·4H_2_O), sodium carbonate (Na_2_CO_3_), phosphate buffered saline (PBS) pH 7.4, glycine, and sodium borohydride (NaBH_4_) were purchased from Sigma-Aldrich (Munich, Germany). Ethylene diamine tetra-acetic acid (EDTA) was purchased from Fluka (Buchs, Switzerland). Sterile NaCl solution, 0.9% was purchased from Fresenius Kabi Deutschland GmbH (Bad Humburg, Germany). Sodium hydroxide (NaOH) and dimethyl sulfoxide (DMSO) were purchased from Carl Roth GmbH (Karlsruhe, Germany). Human serum albumin solution 20% was purchased from Grifols Deutschland GmbH (Frankfurt, Germany). Sodium bicarbonate (NaHCO_3_) was purchased from RCI Labscan (Bangkok, Thailand). Lipopolysaccharide from *Escherichia coli* O111:B4 (LPS) was purchased from Sigma-Aldrich (Saint Louis, MO, USA). Dulbecco’s Minimum Essential Media (DMEM) and Roswell Park Memorial Institute 1640 Medium (RPMI), penicillin/streptomycin, and fetal bovine serum (FBS) were purchased from Gibco (Grand Island, NY, USA). MTT reagent (3-[4,5-dimethylthiazol-2-yl]-2,5-diphenyl tetrazolium bromide) was purchased from Bio Basic (Markham, ON, Canada).

### 2.1. Preparation of Non-Sericin Loaded Human Serum Albumin Micro Particles (NS-HSA MPs)

**Figure 1:**
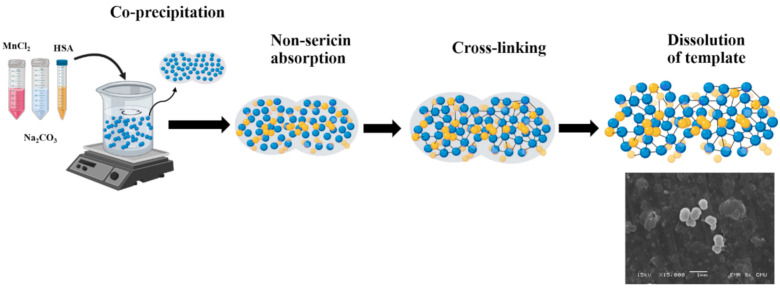
Fabrication procedure of non-sericin loaded human serum albumin micro particles (NS-HSA MPs) using the coprecipitation crosslinking dissolution technique (CCD-technique).

Non-sericin compounds (NS) were obtained from silk cocoon by extraction using ethanol as a solvent. Non-sericin was extracted by maceration with 95% ethanol at room temperature for 24 h. After that, the extract solution was filtrated and evaporated for solvent removal, and then lyophilized to obtain dry extracts [20]. The NS-HSA-MPs were fabricated based on the CCD technique [19] with some modifications. Briefly, 0.125 M MnCl_2_ and 10 mg/mL HSA were mixed. Then, 0.125 M Na_2_CO_3_ was rapidly added under stirring at a stirring speed of 1500 RPM for 30 s at room temperature to produce HSA-MnCO_3_-MPs. After that, 0.05% HSA was added to the mixture for 5 min while being stirred to avoid agglomeration in the particles. The HSA-MnCO_3_-MPs suspension was washed three times with 0.9% NaCl solution by centrifugation at 3000× *g* for 5 min and then resuspended in 0.9% NaCl solution. For loading of NS into particle, NS was dissolved in 100% DMSO at a volume ratio of 1:1. Then, NS solutions with concentrations of 1.25, 2.5, 5, 7.5, and 10 mg/mL were added into the HSA-MnCO_3_-MPs. The suspension was incubated on a rolling mixer for 1 h at room temperature. After that, the suspension was washed three times with 0.9% NaCl. Subsequently, 0.1% glutaraldehyde (GA) was added into the suspension as a cross-linker and then incubated for 1 h at room temperature. Next, 0.08 M glycine and 0.625 mg/mL NaBH_4_ were added into the suspension in order to quench the remaining GA for 30 min at room temperature. Finally, MnCO_3_ templates were dissolved by adding 0.25 M EDTA with incubation time of 30 min at room temperature. The resulting particles were washed three times with 0.9% NaCl solution by centrifugation at 10,000× *g* for 10 min and then resuspended in 0.9% NaCl for further use. The scheme of the fabrication procedure is shown in Figure 1.

### 2.2. Characterization of Non-Sericin Loaded Human Serum Albumin Micro Particles (NS-HSA MPs)

#### 2.2.1. Size, Polydispersity Index (PDI), and Zeta Potential (ZP) of NS-HSA MPs

NS-HSA MPs were resuspended in phosphate buffered saline (PBS) (Sigma aldrich, Munich, Germany) with a pH value of 7.4. The size and polydispersity of the NS-HSA MPs were determined using a zetasizer nano instrument (Malvern Instruments Ltd., Malvern, UK). Additionally, the zeta potential of the particles was investigated. All results were expressed as mean ± standard deviation.

#### 2.2.2. Human Serum Albumin Micro Particles (HSA-MPs) and Non-Sericin Loaded Human Serum Albumin Micro Particle (NS-HSA MPs) Morphology

The particles were observed under confocal laser scanning microscopy (CLSM) (Zeiss LSM 510 Meta, Zeiss MicroImaging GmbH, Jena, Germany) with a 100× oil-immersion objective. The CLSM system was performed at an excitation wavelength of 488 nm and an image of the emitted fluorescence was obtained at a wavelength of 505 nm. The size of particles was analyzed by using ImageJ-1 software (NIH, Bethesda, MD, USA). Furthermore, the surface morphology and the size distribution of the NS-HSA MPs were also investigated using a scanning electron microscope (SEM) (JEOL, JSM-5910LV Peabody, MA, USA). The samples were prepared by applying a drop of the particle suspension on cupper tape and dried overnight. Then, the samples were coated with gold before observation.

### 2.3. Entrapment Efficiency of Non-Sericin Loaded Human Serum Albumin Micro Particles (NS-HSA MPs)

#### 2.3.1. Entrapment efficiency of NS-HSA MPs

HSA MPs were incubated with 100% DMSO for 1 h in a rolling mixture at room temperature followed by centrifugation at 10,000× *g* for 10 min. The supernatant was collected, and the sediment was resuspended in DMSO. This process was repeated until the supernatant solution became clear. The concentrations of free NS in the supernatants were measured by the determination of absorbance using a UV-vis-spectrophotometer (Hitachi U2800, Hitachi High-Technologies Corporation, Tokyo, Japan) at wavelengths of 200–700 nm. The percentage of entrapment efficiency (%EE) value was calculated according to the following equation:

$$\%\;\mathrm{EE}=\frac{\mathrm{Amount}\;\mathrm{of}\;\mathrm{extract}\;\mathrm{in}\;\mathrm{particle}\;\left(\mathrm{mg}\right)}{\mathrm{Total}\;\mathrm{amount}\;\mathrm{of}\;\mathrm{extract}\;\left(\mathrm{mg}\right)}\times 100$$

#### 2.3.2. Fourier Trnsformed Infrared Spectroscopy (FTIR) Study

NS-HSA MPs were characterized by FTIR technique to obtain the FTIR spectra from chemical groups of the NS extract and NS-HSA MPs. The NS-HSA MPs and HSA MPs were prepared as liquid samples with deionized water, while NS extract was prepared in DMSO. Then, the sample were filled into the pathlength cell of Nicolet instrument. Subsequently, the FTIR spectra were recorded in the mid-IR region of 4000–500 cm^−1^ with a spectral resolution of 4 cm^−1^ (Thermo Scientific, NICOLET 6700, Waltham, MA, USA).

### 2.4. Uptake of Non-Sericin Loaded Human Serum Albumin Micro Particle (NS-HSA MPs) in A549 Cell Line

#### 2.4.1. Cultivation of Cell Line

Human lung adenocarcinoma A549 cell line, kindly provided by Prof. Sergio Moya (CIC biomaGUNE, San Sebastian, Spain), was used for the investigation of NS-HSA MPs cellular uptake. The A549 cell line was cultured in RPMI 1640 medium supplemented with 1% penicillin/streptomycin and 10% (*v*/*v*) heat inactivated FBS. Cells were cultured until confluence was reached in the humidified 5% CO_2_ atmosphere of an incubator maintained at 37 °C.

#### 2.4.2. Analysis of Cellular Uptake by Flow Cytometric Analysis

NS-HSA MPs (1%), 200 µL and HSA MPs (1%), 200 µL were labeled with fluorescent dye after being cross-linked and quenched with glutaraldehyde by incubation with 2 mg/mL of FITC and 1 M NaHCO_3_ solution at room temperature in a light protection case for 1 h. After that, the particles with FITC were dissoluted with 0.25 M EDTA for 30 min at room temperature followed by a washing step with 0.9% NaCl and centrifugation at 10,000× *g* for 10 min.

NS-HSA MPs and HSA MPs labeled with FITC at 1000 and 5000 particles/cell were added to A549 cells and they were incubated for 24 h. The cells were then harvested, washed with PBS buffer, and fixed with 4% paraformaldehyde. After that, the percent-age of cell uptake was analyzed using flow cytometry (FACS-Canto II, Becton and Dickinson, Franklin Lakes, NJ, USA) in the APC channel.

#### 2.4.3. Determination of Cellular Uptake by Confocal Laser Scanning Microscopy (CLSM)

A549 cells were seeded in a 24-well tissue culture plate at a concentration of 1 × 10^5^ cells/well and allowed to form a confluent monolayer for 24 h in a humidified 5% CO_2_ incubator at 37 °C. After that, media were discarded and replaced with RPMI media containing HSA MPs or NS-HSA MPs labeled with FITC. The A549 cell line was incubated with FITC labeled HSA MPs and NS-HSA MPs of 1000 and 5000 particles/cell for 24 h. After incubation for 24 h, the cells were washed with PBS buffer three times and fixed with 4% paraformaldehyde for 10 min. The degree of cell uptake was then determined by confocal microscopy at 488 nm.

#### 2.4.4. Analysis of Three-Dimensional Localization of Particles by 3D Holotomography (HT) Microscopy

Three-dimensional localization of FITC labeled particles in the cells was analyzed by HT microscopy (3D HT-2 system, Tomocube, Daejeo, Korea) after incubation with the A549 cell line for 24 h. The cells were then harvested and fixed with paraformaldehyde. The 3D images of the particles inside the cells were observed under an HT microscope.

### 2.5. Effect of Particles on Macrophage Stimulation

The RAW 264.7 macrophage cell line was used in this study, while stimulation of the RAW 264.7 macrophage response from NS-HSA MPs and HSA MPs was determined by the release of nitric oxide in vitro. Cell metabolic activity was performed on the macrophage cells using MTT assay. The particles 100, 1000, and 5000 particle/cell were chosen for stimulation of RAW 264.7 macrophage cells using nitric oxide (NO) assay [21]. In brief, macrophage cells were cultured in DMEM medium supplemented with 10% FBS at 37 °C in a 5% CO_2_ atmosphere. After cell adherence, cells were treated with each concentration of NS-HSA MPs, HSA MPs and 1 µg/mL of LPS for 24 h. Nitric oxide production was then investigated from NS-HSA MP and HSA MP stimulated macrophage cells by comparing them to LPS stimulated cell control. After the supernatant from the stimulated cells was collected, nitric oxide was analyzed in the form of nitrites using the Griess reaction assay. Colorimetric reactions were determined using a microplate reader at 540 nm, while sodium nitrite (Cambourne, Cambridge, UK) was used as a positive control.

### 2.6. Statistical Analysis

The experiments were performed in triplicate and data were expressed as mean ± SD values. Statistical significance was evaluated by unpaired Student’s *t*-tests or one-way ANOVA. The level of significance was established at *p* < 0.05.

## 3. Results and Discussion

### 3.1. Particle Preparation 

**Figure 2:**
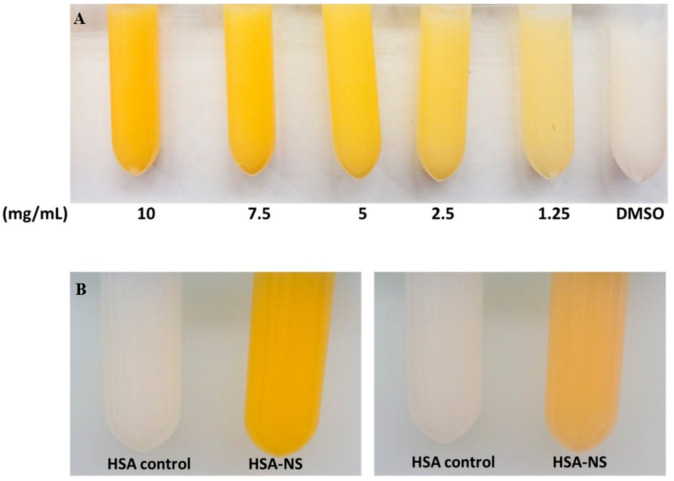
Non-sericin loaded human serum albumin micro particles (NS-HSA MPs) by CCD-technique. Non-sericin components were varied at different concentrations to increase the percentage of entrapment efficiency (**A**). Non-sericin samples were incubated (**B**) before the cross-linking step (left) and after the cross-linking step (right).

NS-HSA-MPs were successfully fabricated using the modified CCD technique (Figure 1). After dissolution the particle template, the final particle suspension of NS-HSA MPs exhibited a yellow color as compared to the white color of HSA-MPs that were used as a control. This indicated that NS was adsorbed into porous structure of the HSA-MnCO_3_ particles (Figure 2). This entrapment was attributed due to hydrophobic interactions that occur between lipophilic substances of non-sericin components with the N-H functional group of albumin proteins. It has been previously shown that the β-carotene compound is bound to the pyrrolidine ring of proline rich zein proteins [22,23], which are supported by NS entrapment into HSA-MnCO_3_. This has occurred since the NS compounds are mostly composed of hydrophobic molecules that possess the same structure as the β-carotene compound.

**Figure 3:**
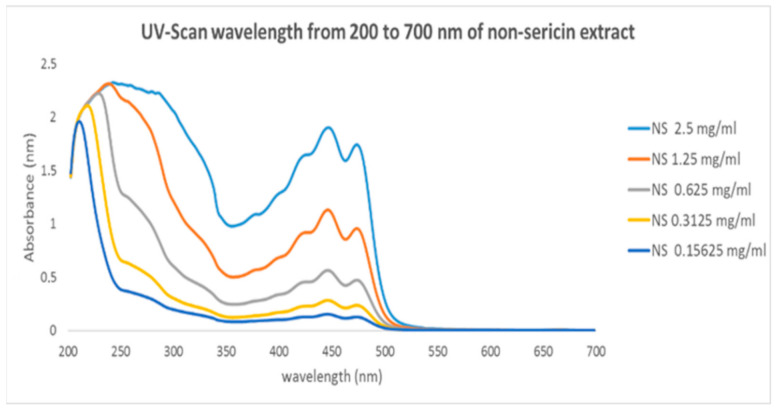
UV-visible spectra of non-sericin extracts at various concentrations in DMSO demonstrating spectra in a range of 200–700 nm.

The UV spectra of the NS extract solution at various concentrations were detected in a range of 200–700 nm, and it was found that there were three main absorbance peaks at 436, 460, and 488 nm (Figure 3) at each NS extract concentration. These findings are in accordance with the results from Zhu and Zhang [24]. They investigated pigment composition in silk cocoons using UV-visible spectroscopy. The main absorbance peaks were found to be in the wavelength range from 400 to 500 nm. Moreover, these compounds displayed UV-Vis spectrum, characteristic for carotenoids [3]. Therefore, three main peaks of the non-sericin extract were composed of carotenoid compounds and could be used to generate a calibration curve of encapsulation efficiency.

In this study, the adsorption of NS to HSA-MnCO_3_ particle varied and depended on the concentration and fabrication steps. These results indicated that non-sericin substance with poor water solubility can be entrapped into the HSA-MnCO_3_ particle via electrostatic and hydrophobic interactions which is similar to the results of doxorubicin loaded HSA-MnCO_3_ particle [16]. The HSA-MnCO_3_ templates were incubated with different concentrations of NS. This is summarized in Table 1. NS extract at 5.0 mg/mL showed the optimum percentage of entrapment efficacy (EE). Therefore, the adsorption of NS into the particles might be dependent on the ratio of particle templates and NS concentration that is available for adsorption. As we know from a previous study involving fabricated particles by using the CCD-technique, MnCO_3_ particle template displayed the potential to entrap poor water solubility such as riboflavin compound and these particles can carry an amount of riboflavin in water four times higher than the solubility of just riboflavin alone [18]. Furthermore, in a study involving curcumin as a hydrophobic molecule, it was found to be capable of being entrapped in the hydrophobic cavity of HSA nanoparticles [25].

The particles were further investigated by a variation of adsorption steps based on the results obtained from various NS concentrations. This was done to achieve the highest concentration of NS in the particles. The HSA-MnCO_3_ particles were incubated with NS-DMSO solution once before and once after the cross-linking step with glutaraldehyde. The results indicated that incubation of the NS extract with particles before the cross-linking step displayed significantly higher adsorption capabilities than incubation of the NS extract with particles after being cross-linked with glutaraldehyde at approximately 43% (Table 1). Therefore, NS could bind to the albumin binding pocket or other hydrophilic binding site. The particles that were cross-linked by glutaraldehyde displayed the strongest capturing capability when compared to the NS incorporated after being cross-linked. The effects of this interaction could be due to the conformation change of HSA by self-polymerization between HSA and NS compounds. Moreover, the aldehyde group of glutaraldehyde reacted with the amino groups of proteins that were mostly associated with lysine and aromatic acids. This interaction had been previously reported in a study involving bovine hemoglobin microparticles [26,27]. In addition, Tazhbayev et al. [28] demonstrated that a urea and cysteine mixture could also be used for cross-linking to affect the conformation change of bovine serum albumin.

**Figure 4:**
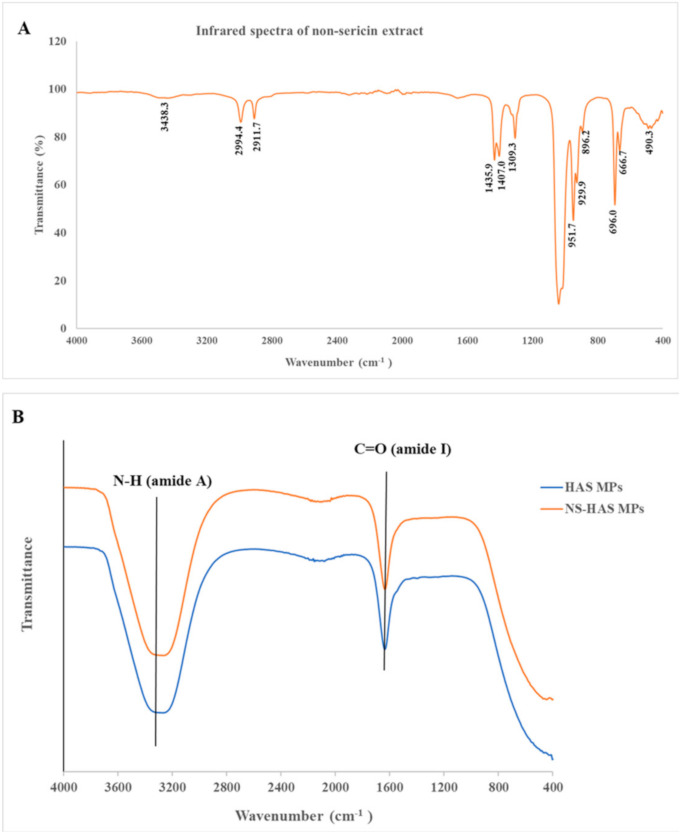
Fourier-transform infrared spectra of non-sericin loaded human serum albumin micro particles, NS-HSA MPs (**A**) and human serum albumin micro particles, HAS-MPs (**B**). The spectra data detected from the 400–4000 cm^−1^ region of non-sericin components incorporated with both the specified particles and the empty particles display a similar pattern.

Additionally, the efficiency of NS encapsulation was observed using FTIR to confirm the incorporation of NS in the MPs. This technique had been previously used to evaluate conformational changes when the particles interact with a substance. The results of the FTIR analysis are shown in Figure 4. The adsorption of the NS extract showed a C-H region stretching in the alkanes group between 3000 and 2840 cm^−1^ and C-H stretching in the aromatic alkenes group between 3100 and 2900 cm^−1^ (NS extract at regions of 2911.7 and 2994.4 cm^−1^). In the regions of 1465–1150 cm^−1^, the asymmetrical C-H bending of CH_3_ of the alkanes was recorded in the NS extract at 1407.0 and 1435.9 cm^−1^. The region of 1309.3 was indicated by methylene twisting and wagging vibrations recorded within the region of 1350–1150 cm^−1^. The bands were observed at 896.2, 929.9, and 951.7 cm^−1^, which referred to the C-C stretching of alkanes between 1200 and 800 cm^−1^. In addition, the stretching of 666.7, 696.0, 896.3, 929.9, and 951.7 cm^−1^ out of the plane C-H bending of the alkenes, alkynes, and aromatic compound groups were recorded between the regions of 1000–650 cm^−1^, 975–600 cm^−1^, and 900–600 cm^−1^, respectively.

Moreover, the spectra of O-H stretching vibration that was related to the alcohol group at 3438.3 cm^−1^ revealed a weak degree of absorption [29]. The functional groups of NS-HSA MPs and HSA MPs occurred during the stretching of the C=O region due to the amide I group at 1636 and 1635 cm^−1^, which represented the major group of albumin proteins. Similarly, the adsorption spectra of the bovine serum albumin for the amide I group were between 1700 and 1600 cm^−1^, which indicated that the proteins served as secondary structural components [30]. The absorption of the bands assigned to N-H stretching vibrations at the regions of 3265 and 3266 cm^−1^ were related to the amide A group [31,32]. Therefore, the spectra of the NS loaded HSA MPs and the empty HSA MPs displayed almost the same characteristic peaks. The results of the FTIR data also suggested that the non-sericin component was cross linked and entrapped to the HSA matrix. This would then confirm that the HSA MPs prepared by the CCD technique had a strong ability to effectively entrap hydrophobic substances in the particles. Previous results have also reported on the encapsulation of fisetin in the HSA nanoparticle using FTIR spectroscopy [31].

### 3.2. Characterization of Non-Sericin Loaded Human Serum Albumin Micro Particles (NS-HSA MPs)

After dissolution of the MnCO_3_-template, the size and zeta potential of NS-HSA MP, and HSA-MP were analyzed by zetasizer (Table 2). NS-HSA MPs exhibited a submicron size ranging from 0.8 to 0.9 µm with a negative zeta potential. The values of the zeta potential of the particles were different depending on the ionic strength of the suspension media such as those composed of water, PBS buffer, and 0.9% NaCl [33]. In accordance with this observation, the size and zeta potential of NS-HSA MPs were not significantly different from the empty HS-MPs when measured in PBS buffer. Moreover, a previous fabrication of hemoglobin particles was based on the coprecipitation of hemoglobin with MnCO_3_ and on the adsorption of HSA with a size of around 600–800 nm [17,19]. Moreover, the establishment of a hemoglobin particle was modified and embedded with polydopamine, which was also identified in the submicron size. The particles displayed excellent biocompatibility and were capable of protecting against the cytotoxic effects that can be caused by polydopamine substances [33].

The size of NS-HSA MPs was investigated by CLSM images. HSA MPs and NS-HSA MPs images are shown in supplementary material (Figure S1). Both particles demonstrated a submicron size with a weak level of autofluorescence from the glutaraldehyde. Generally, the size of the particles depends upon certain precipitation factors such as the type and concentration of the salt, temperature, pH, mixing speed, reaction time, and any additives [18,19].

**Figure 5:**
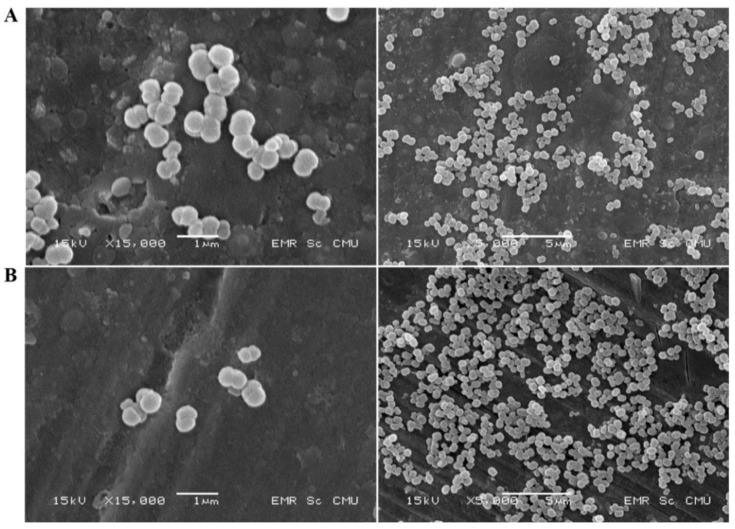
SEM images of non-sericin loaded human serum albumin micro particles, HSA-MP (**A**), and human serum albumin micro particles, NS-HSA-MP (**B**).

Moreover, the morphology of both particles was evaluated by SEM microscope. The particles from our formulation displayed a peanut-like shape (Figure 5) and were almost uniform in morphology with narrow size distribution. The shape was similar to the findings of a previous report on MnCO_3_ particles that involved coprecipitation with hemoglobin, in which the particles appeared as a peanut shape and displayed a rougher surface than the CaCO_3_ particles [15,19,33].

### 3.3. Determination of Cellular Uptake of NS-HSA MPs and HSA MPs 

#### 3.3.1. FITC-Labeling HSA MPs and NS-HSA MPs

**Figure 6:**
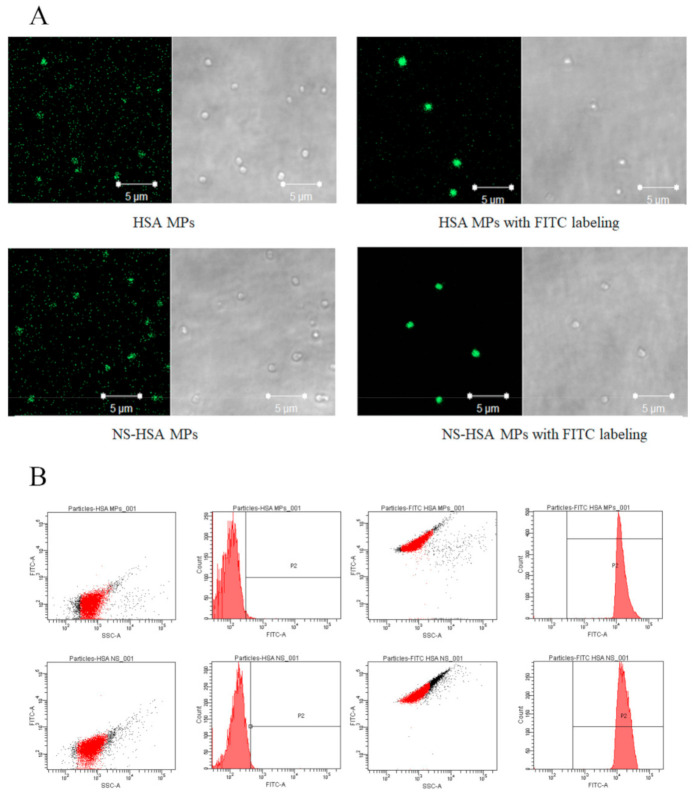
Human serum albumin micro particles, HSA-MPs, and non-sericin loaded human serum albumin micro particles, NS-HSA-MPs, after FITC labeling and nonlabeling indicated that the fluorescent particles were higher than the nonlabeled particles after observed by CLSM (**A**) and after investigated by FACS (**B**).

Initially, HSA MPs and NS-HSA MPs were labeled with fluorescein isothiocyanate (FITC) for the investigation of cellular uptake. The labeled particles were determined by CLSM and FACS analysis. According to the CLSM results, there were no differences shown in size between the particles labeled with FITC and the nonlabeled particles (Figure 6A). Nonlabeled MPs exhibited only autofluorescence and the fluorescence intensity was much lower in comparison with the FITC labeled MPs. Flow cytometry was used to confirm the degree of fluorescence intensity indicating that the FITC fluorescent intensity increased by almost 100% of the labeled particles. Mean fluorescence intensity (MFI) values of labeled MPs and nonlabeled MPs were 8504.00 ± 32.53 and 498.00 ± 4.24, respectively (Figure 6B). Moreover, the results of the size distribution and zeta potential of the particles after being labeled with FITC were not significantly different from those of the nonlabeled particles when analyzed by zetasizer (Table 3).

The FITC labeled HSA was further investigated in terms of A549 cell line uptake. The findings of this investigation were similar to those of a previous study that showed that conjugated FITC-HSA was attached to the A549 cells via the albumin receptor [34]. FITC was conjugated with proteins under alkaline conditions, while the amino acid group of lysine was found to be the most probable site of conjugation. Moreover, the fluorescent labeling nanoparticles facilitated the rapid detection and a high degree of sensitivity in quantifying cell-associated nanoparticles [35], while FITC exhibited a low effect on protein/peptide biological activity [36]. Therefore, it was confirmed that HSA MPs that were labeled with FITC could be used in cellular uptake experiments.

#### 3.3.2. Determination of Cellular Uptake of NS-HSA MPs by Flow Cytometry

**Figure 7:**
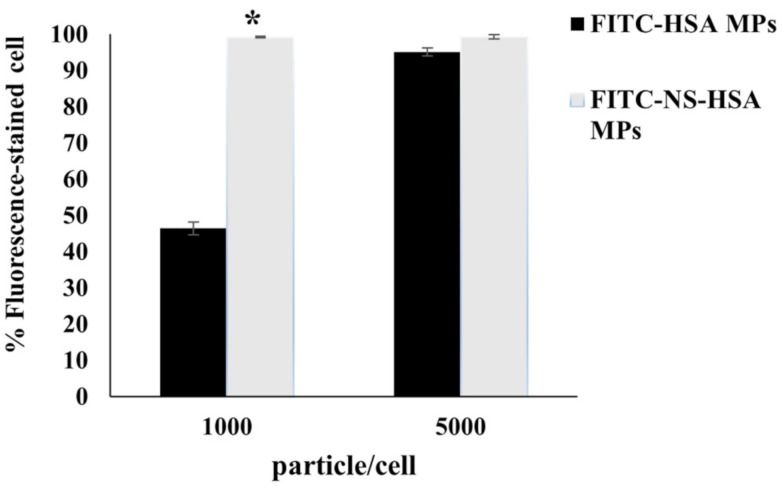
Percentage of cellular uptake of FITC- NS-HSA MPs and FITC- NS-HSA MPs by A549 cells analyzed by the presence of fluorescence intensity by FACS. Data are presented as mean ± SD. * *p* ≤ 0.05 was determined by *t*-test.

The cellular uptake of FITC labeled HSA MPs and NS-HSA MPs in the A549 cell line were analyzed by flow cytometry. A549 cells were used in the cellular uptake model due to the potential benefit of albumin receptors on the cell surface [37]. The percentage of cells which contain fluorescent labeled HSA or NS-HSA MPs was determined after incubation of A549 cell line with MP at concentrations of 1000 and 5000 MPs/cell for 24 h (Figure 7). The results of the cellular uptake analysis indicated that particles at a concentration of 1000 particles per cell displayed the percentage of fluorescence-stained cells of HSA MPs and NS-HSA MPs at 46.43 ± 1.72% and 99.17 ± 0.23%, respectively. Meanwhile, when the particles were taken up at 5000 particles per cell, the percentage of fluorescence-stained cells increased to 95.13 ± 1.06 and 99.30 ± 0.53% in HSA MPs and NS-HSA MPs, respectively. Therefore, the NS-HSA MPs at a low concentration of 1000 particles per cell demonstrated the highest level of fluorescence-stained cell. However, at a low concentration of HSA-MPs, a significantly lower mean fluorescence intensity value was recorded. Therefore, NS-HSA MPs were recognized and taken up into the A549 cells. Furthermore, the internalization of MPs in the cells was verified and confirmed using a confocal laser scanning (CLSM) microscope and 3D Holotomography (HT) microscopy.

#### 3.3.3. Determination of Cellular Uptake of NS-HSA MPs by Confocal Laser Scanning microscope (CLSM)

**Figure 8:**
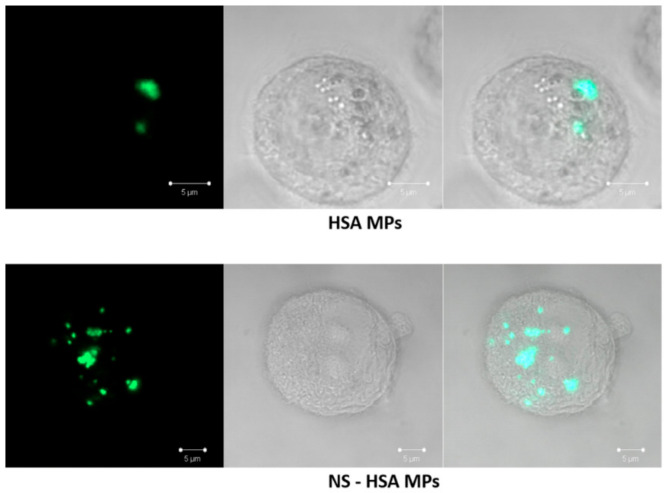
Uptake of FITC labeled HSA MPs and NS-HSA MPs inside A549 cells after incubation for 24 h. Each particle was detected in the Z-stacks pattern under a confocal laser scanning microscope (CLSM) with fluorescence, bright field, and merging image, respectively.

The internalization of the particles in cells was investigated by CLSM microscope. The CLSM microscope was set at the Z-stacks pattern for the multilayer capturing of the cells. The results showed that both HSA MPs and NS-HSA MPs labeled with FITC were internalized into the cells (Figure 8). In general, internalization of protein-based particles can occur through endocytosis by recruiting the dynamin and clathrin-dependent pathways [38]. A recent report by Yumoto [37] demonstrated that FITC-labeled albumin particles were taken up in A549 cells by a low-affinity system that was mediated by the endocytosis and clathrin-mediated endocytosis pathways. This could be suggestive of a common uptake mechanism of the albumin receptor. Similarly, a study involving doxorubicin revealed that it was loaded into HSA-MPs, uptaken by the A549 cells and then localized in the cell lysosomal compartment [16].

#### 3.3.4. Cellular Uptake of NS-HSA MPs by 3D Holotomography (HT) Microscopy

**Figure 9:**
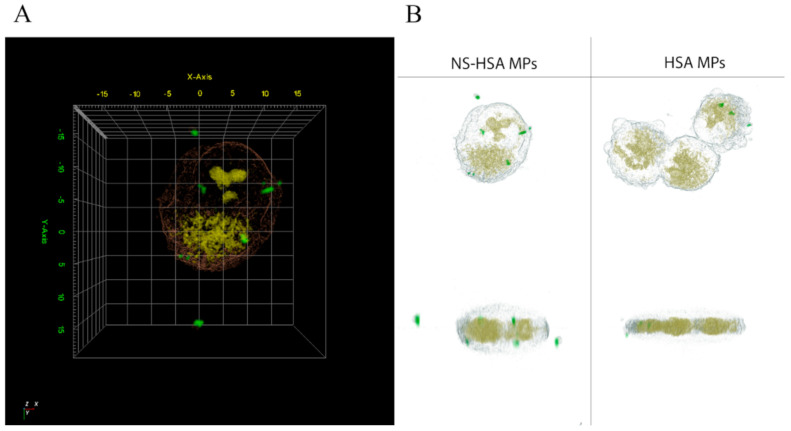
3D RI tomogram of NS-HSA MPs (**A**) and HSA MPs that were internalized in A549 cells by fluorescence imaging together with RI imaging (**B**).

Holotomography and 3D fluorescence imaging was also used to analyze the particles entering into the cells. The results verified that the degree of fluorescent intensity of NS-HSA MPs and HSA MPs labeled with FITC was located inside the cells (Figure 9A,B). The refractive index (RI) value of the A549 cell membrane was found to be constant from 1.342 to 1.351 RIU (refractive index unit). Furthermore, the 3D distributions depended upon the refractive index (RI) value in the individual organelle or cell. The RI value was expressed as unique information with regard to the local proteins located inside the cells and through the monitoring of cell morphology. Moreover, 3D fluorescence imaging of gold nanoparticles inside the cell was also observed in HeLa and 4T1 cells [39]. Previous research involving 3D images utilized quantitative images to measure dynamic changes of lipid droplets in live cells, Huh-7. The quantity and mass of lipid droplets in the cellular compartment was measured by RI distribution [40]. Thus, the particles were detected in A549 cells by using RI distribution in combination with 3D tomograms and the 3D fluorescence imaging method [41].

### 3.4. Effect of Particles on Macrophage Stimulation

**Figure 10:**
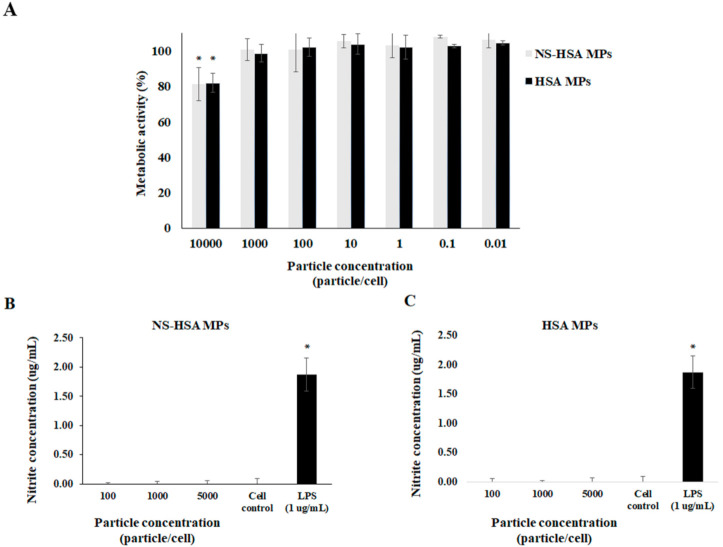
Metabolic activity of RAW264.7 cells after treatment with NS-HSA MPs and HSA MPs (**A**) and stimulation of macrophage to release nitric oxide by NS-HSA MPs (**B**) and HSA MPs (**C**). Data are presented as mean ± SD. * *p* ≤ 0.05 was determined by *t*-test.

The metabolic activity of RAW264.7 macrophage cells was evaluated by MTT assay. NS-HSA MPs and HSA MPs at the particle concentration lower than 1000 particle/cell induced no changes of metabolic activity on RAW macrophages (Figure 10A). After RAW264.7 macrophage cells were stimulated with three concentrations (100, 1000, 5000 particle/cell) of NS-HSA MPs and HSA MPs, the release of nitric oxide (NO) was not detected. The results indicated that both particles could not promote NO production, which was the same for the non-LPS treated cell control. The effects towards LPS induction exhibited NO release at approximately 1.78 ± 0.28 µg/mL (Figure 10B,C). Consequently, HSA MPs might be suitable for use as a biopolymer carrier since albumin protein was neither toxic nor stimulation of inflammation to the cells since NS-HSA MPs and HSA MPs did not stimulate NO release from macrophage cells.

## 4. Conclusions

Hydrophobic non-sericin compounds obtained from ethanolic silk cocoon extracts were encapsulated into human serum albumin protein particles that were generated from the MnCO_3_ particle template using the CCD-technique. The entrapment efficiency of the non-sericin extract was highly effective for entrapment after incubating the extract with the particle template before being cross-linked. The resulting particles revealed particle-size distribution of the submicron size with a negative charge. Moreover, NS-HSA MPs were internalized into the A549 cell line. Interestingly, NS-HSA MPs and HSA-MPs have no capacity to stimulate nitric oxide production in RAW264.7. These findings suggested that HSA MP could be considered as a carrier molecule to enhance the ability of insoluble substances, as well as in the incorporation of other natural compounds for the enhancement of a range of biological functions.

## Figures and Tables

**Table 1:** Percentage of entrapment efficiency of non-sericin incorporation into HSA MPs.

Absorbance	Entrapment Efficiency * (%)		
	436 nm	460 nm	488 nm
Concentration of			
NS (mg/mL)		57.77 ± 6.25	57.60 ± 7.56
1.25	54.47 ± 5.59	44.59 ± 1.71	44.44 ± 2.21
2.5	43.48 ± 3.03	42.28 ± 7.14	43.65 ± 6.17
5.0	42.28 ± 7.14	38.68 ± 4.81	39.34 ± 5.27
7.5	33.87 ± 4.86	32.35 ± 1.38	32.19 ± 1.60
10.0	30.30 ± 0.34		
NS 5 mg/mL incubation step		42.97 ± 6.28	43.65 ± 6.17
before cross-linking	42.28 ± 7.14	7.60 ± 1.43	7.31 ± 1.12

* Data are presented as mean ± SD. (n ₌ 3).

**Table 2:** Characteristics of hydrodynamic diameter and zeta potential in NS-HSA MPs and HAS-MPs by zetasizer.

Particle	Size *		Zeta Potential *		
	Z-Average Size (nm)	PDI	ZP (mV)	Mob (µmCm/Vs)	Cond (mS/cm)
NS-HSA MPs	918.50 ± 55	0.21 ± 0.04	−14.81 ± 0.51	−1.14 ± 0.04	17.38 ± 0.58
HSA MPs	893.57 ± 48	0.19 ± 0.06	−14.36 ± 0.38	−1.10 ± 0.03	17.38 ± 0.60

* Data are presented as mean ± SD. (n = 6); Zeta potential (ZP), electrophoretic mobility unit (Mob), and conductivity (Cond).

**Table 3:** Characteristics of hydrodynamic diameter and zeta potential after NS-HSA MPs and HSA MPs labeling with FITC by zetasizer.

Particle FITC Labeling	Dilution Solution	Size *		Zeta Potential *		
		Z-Average Size (nm)	PDI	ZP (mV)	Mob (µmCm/Vs)	Cond (mS/cm)
NS-HSA MPs	PBS pH7.4	813 ± 70	0.39 ± 0.07	−14.73 ± 0.24	−1.13 ± 0.02	17.33 ± 0.58
HSA MPs	PBS pH7.4	901 ± 60	0.18 ± 0.01	−14.89 ± 0.97	−1.15 ± 0.08	17.41 ± 0.01

* Data are presented as mean ± SD. (n = 3); Zeta potential (ZP), electrophoretic mobility unit (Mob), and conductivity (Cond).

## Data Availability

The data created in this study are fully depicted in the article and supplementary material.

## Supplementary Materials

The following material is available online at https://www.mdpi.com/2073-4360/13/3/334/s1, Figure S1: Confocal laser scanning images showed human serum albumin micro particles, HSA-MP and non-sericin loaded human serum albumin micro particles, NS-HSA-MP, by fluorescence intensity (left) and transmission image (right).
