# Supplementary material for: Fabrication and Characterization of Human Serum Albumin Particles Loaded with Non-Sericin Extract Obtained from Silk Cocoon as a Carrier System for Hydrophobic Substances
Source: Polymers (Basel). 2021 Jan 21;13(3):334. doi: 10.3390/polym13030334 (PMC7865381; doi:10.3390/polym13030334)
Supplement: Supplementary file 1 [file polymers-13-00334-s001.pdf]

polymers

MDPI

Supplementary material

Fabrication and Characterization of Human Serum Albumin Particles Loaded with Non-Sericin Extract Obtained from Silk Cocoon as a Carrier System for Hydrophobic Substances

Kanyaluck Jantakee [1,2], Ausanai Prapan [3], Saranya Chaiwaree [4], Nittiya Suwannasom [5], Waraporn Kaewprayoon [4], Radostina Georgieva [6,7], Yingmanee Tragoolpua [1,8,*] and Hans Bäumler [6,*]

[1] Department of Biology, Faculty of Science, Chiang Mai University, Chiang Mai 50200 Thailand; kanyaluck-jan@gmail.com (K.J.); yingmanee.t@cmu.ac.th (Y.T.)
[2] The Graduate School, Chiang Mai University, Chiang Mai 50200, Thailand; kanyaluckjan@gmail.com
[3] Department of Radiological Technology, Faculty of Allied Health Sciences, Naresuan University, Phitsanulok 65000, Thailand; ausanaip@nu.ac.th
[4] Faculty of Pharmacy, Payap University, Chiang Mai 50000, Thailand; mam.chaiwaree@gmail.com (S.C.); waraporn.kpy@gmail.com (W.K.)
[5] School of Medical Sciences, University of Phayao, Phayao 56000, Thailand; nittiya.su@up.ac.th
[6] Charité, Universitätsmedizin Berlin, Institute of Transfusion Medicine, 10117 Berlin, Germany; radostina.georgieva@charite.de (R.G); hans.baeumler@charite.de (H.B.)
[7] Department of Medical Physics, Biophysics and Radiology, Medical Faculty, Trakia University, 6000 Stara Zagora, Bulgaria; radostina.georgieva@charite.de
[8] Research Center in Bioresources for Agriculture, Industry, and Medicine, Faculty of Science, Chiang Mai University, Chiang Mai 50200, Thailand; yingmanee.t@cmu.ac.th
* Correspondence: yingmanee.t@cmu.ac.th (Y.T.) and hans.baeumler@charite.de (H.B.)

Citation: Jantakee, K.; Prapan, A.; Chaiwaree, S.; Suwannasom, N.; Kaewprayoon, W.; Georgieva, R.; Tragoolpua, Y.; Bäumler, H. Fabrication and Characterization of Human Serum Albumin Particles Loaded with Non-Sericin Extract Obtained from Silk Cocoon as a Carrier System for Hydrophobic Substances. *Polymers* **2021**, *13*, x. https://doi.org/10.3390/xxxxx
Citation: Lastname, F.; Lastname, F.; Last-name, F. Title. *Polymers* **2021**, *13*, x. https://doi.org/10.3390/xxxxx
Academic Editor: Beom Soo Kim

Received: 04 January 2021
Accepted: 16 January 2021
Published: date

Publisher's Note: MDPI stays neutral with regard to jurisdictional claims in published maps and institutional affiliations.

Copyright: © 2020 by the authors. Submitted for possible open access publication under the terms and conditions of the Creative Commons Attribution (CC BY) license (http://creativecommons.org/licenses/by/4.0/).

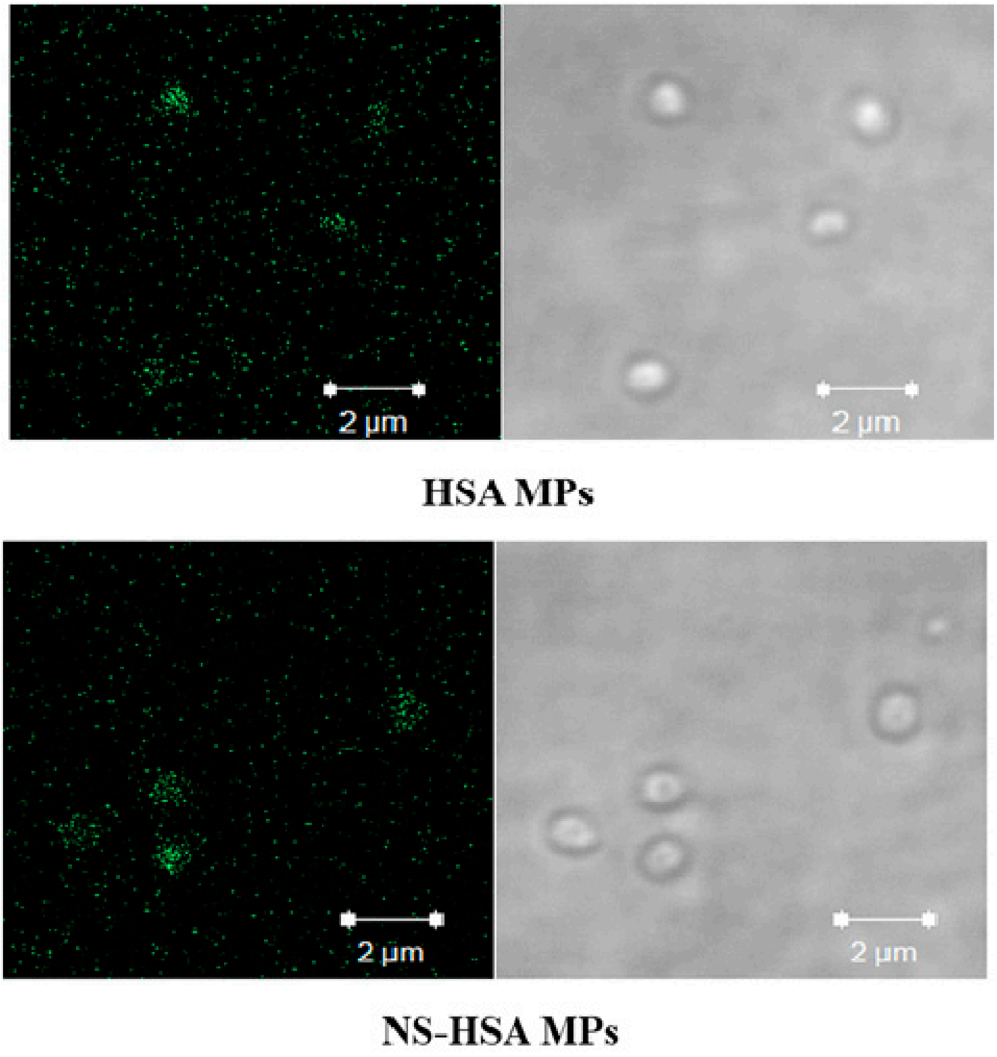

Figure S1. Confocal laser scanning images showed human serum albumin micro particles, HSA-MP and non-sericin loaded human serum albumin micro particles, NS-HSA-MP.
